# Supplementary material for: Hypoxic Environment and Paired Hierarchical 3D and 2D Models of Pediatric H3.3-Mutated Gliomas Recreate the Patient Tumor Complexity
Source: Cancers (Basel). 2019 Nov 26;11(12):1875. doi: 10.3390/cancers11121875 (PMC6966513; doi:10.3390/cancers11121875)
Supplement: Supplementary file 1 [file cancers-11-01875-s001.pdf]

Supplementary Materials

Hypoxic Environment and Paired Hierarchical 3D and 2D Models of Pediatric *H3.3*-Mutated Gliomas Recreate the Patient Tumor Complexity

Anne-Florence Blandin, Aurélie Durand, Marie Litzler, Aurélien Tripp, Éric Guérin, Elisa Ruhland, Adeline Obrecht, Céline Keime, Quentin Fuchs, Damien Reita, Benoit Lhermitte, Andres Coca, Chris Jones, Isabelle Lelong Rebel, Pascal Villa, Izzie Jacques Namer, Monique Dontenwill, Dominique Guenot and Natacha Entz-Werle

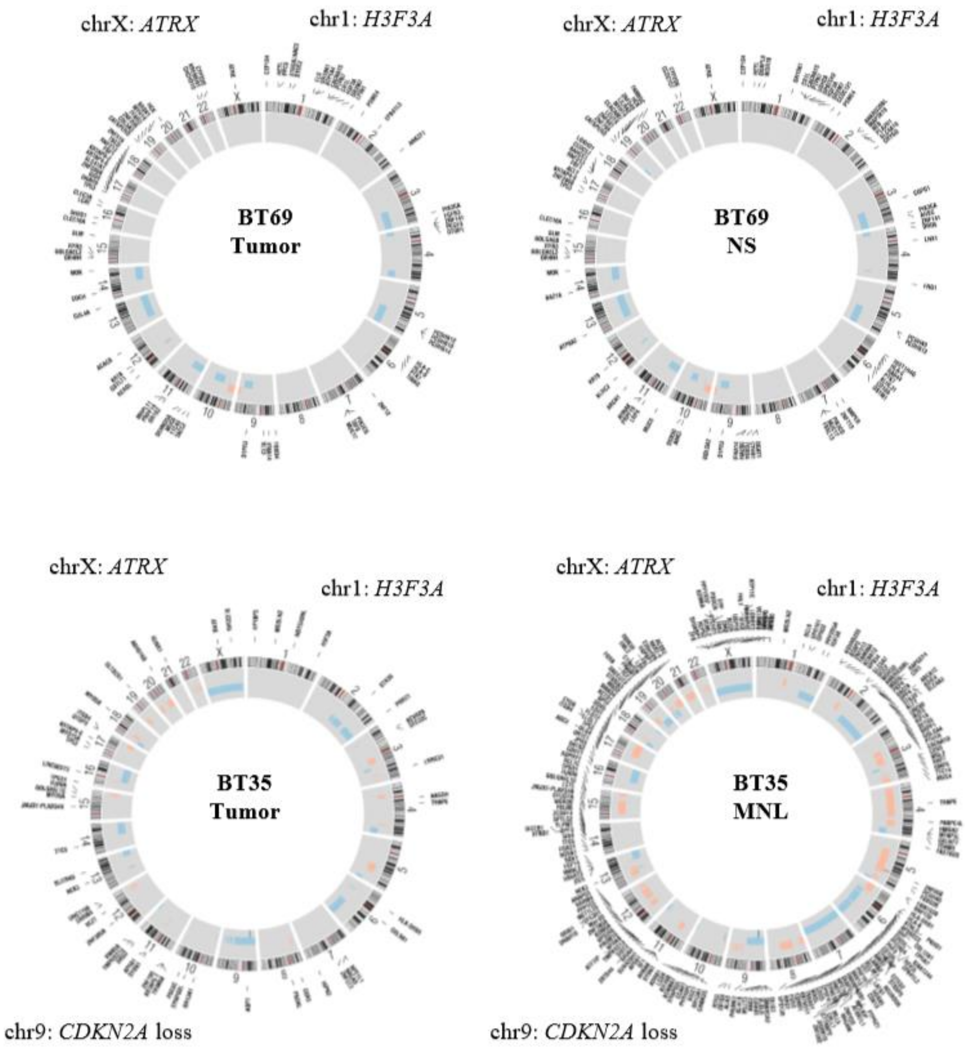

Figure S1. Circos plots depicting genomic aberrations in patient tumors and the paired PDCLs.

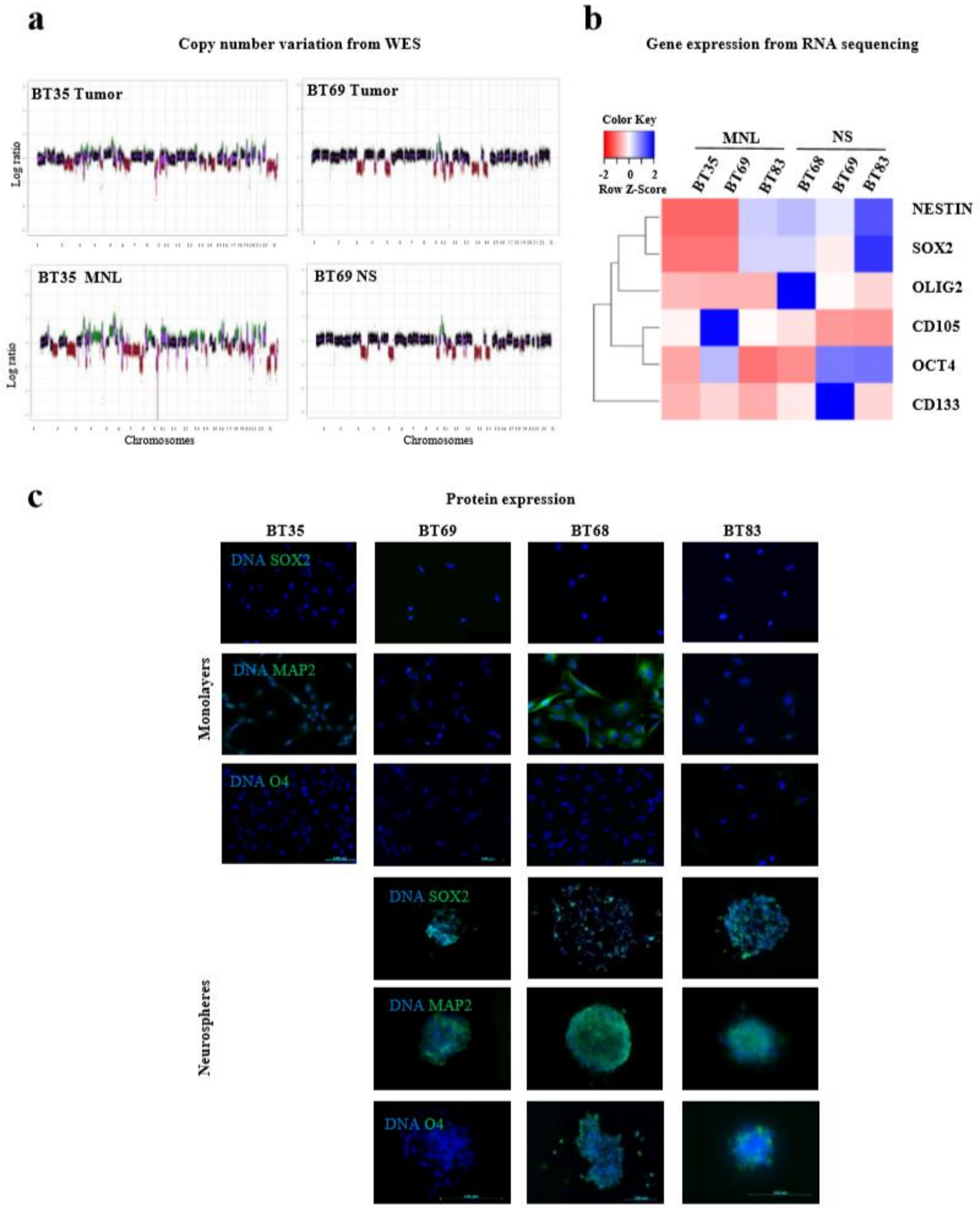

Figure S2. Neurosphere culture might reflect more closely the genomic profile and the lineage markers of high-grade gliomas.

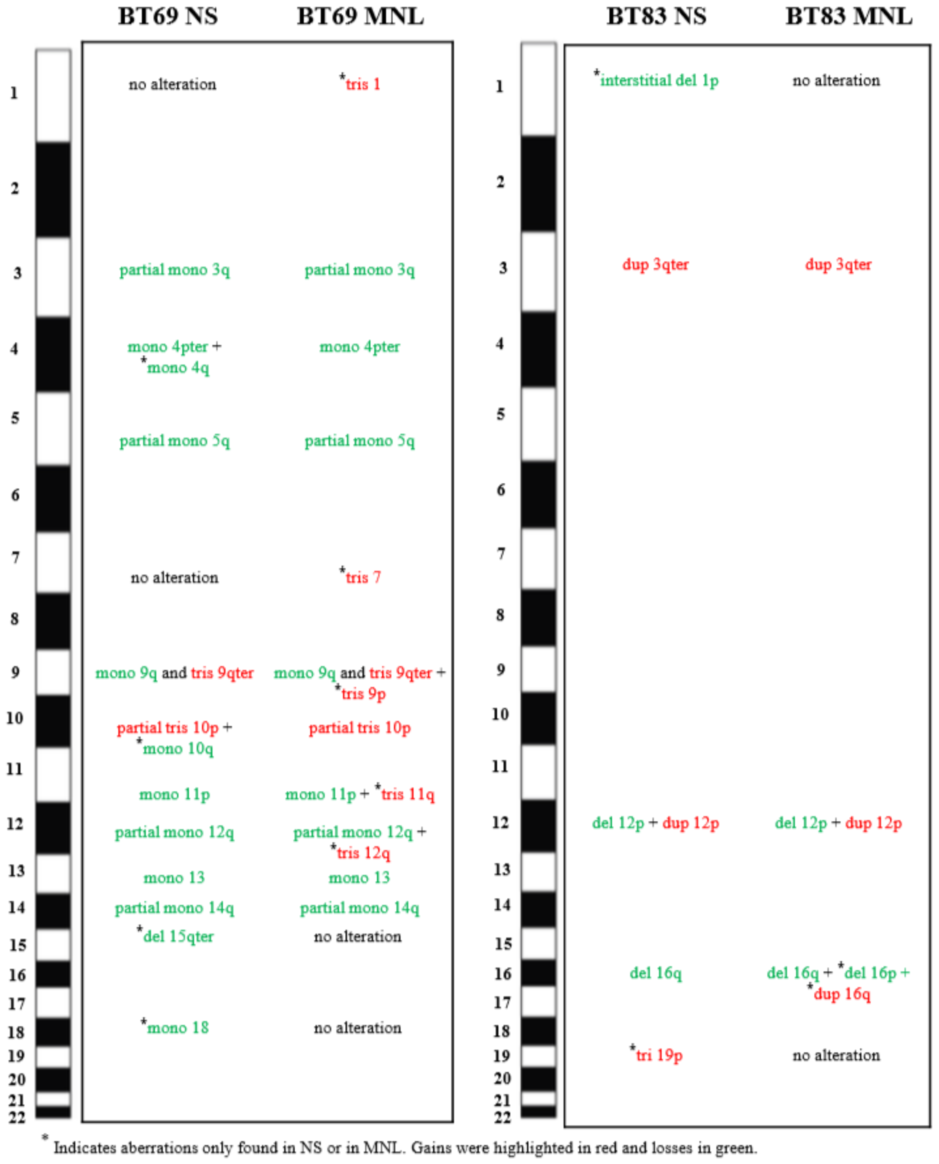

Figure S3. Significant copy number variations between patient-derived neurosphere and the paired-monolayer.

a

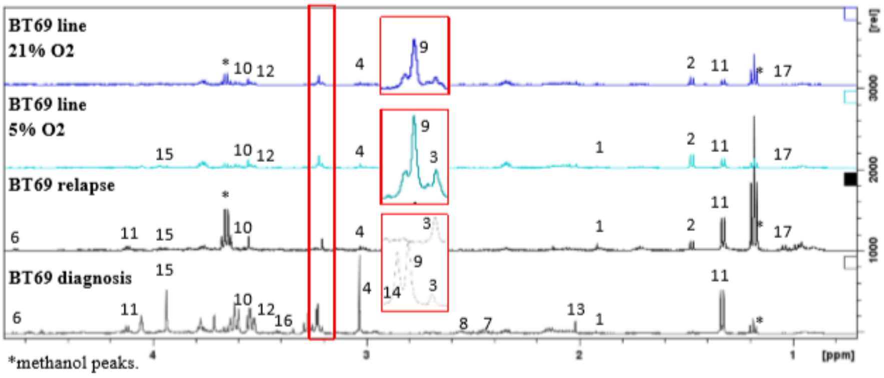

b

	Metabolite assignments			Tumor BT69		PDCL BT69	
	Metabolites	^{1}H chemical shift (ppm)	^{13}C chemical shift (ppm)	diagnosis	relapse	5% O2	21% O2
1	acetate	1.92	25.90	0,12	0,3	0,11	0,06
2	alanine	1.48	18.70	0,26	0,97	1,21	0,47
3	choline	3.22	56.42	0,18	0,17	0,13	0,08
4	Creatine	3.03	39.56	1,23	0,22	0,2	0,11
5	GABA	2.30	36.97	3,43	0,91	0,72	0,38
6	β-Glucose	4.65	98.56	0,36	0,42	0,32	0,38
7	Glutamate	2.34	35.99	1,6	1	2,13	1,31
8	Glutamine	2.45	33.29	1,53	0,38	0,23	0,23
9	GPCho	4.33	62.09	0,901	0,21	0,44	0,337
10	Glycine	3.56	43.99	0,32	0,28	0,15	0,1
11	Lactate	4.12	71.05	3,31	2,29	1,07	0,66
12	Myo-inositol	3.62	74.93	6,48	1,52	1,22	0,83
13	NAA	2.02	24.58	0,36	0,12	0,8	0,12
14	PCho	4.16	60.57	0,85	0,14	0,42	0,3
15	Serine	3.96	62.84	1,41	0,94	1,18	0,9
16	Taurine	3.42	37.93	0,73	0,5	0,21	0,12
17	Valine	1.04	20.65	0,06	0,55	0,11	0,04
	GPCho/Pcho (9/14)			1.06	1.5	1.12	1.04
	Gly/MyoI (10/12)			0.04	0.18	0.12	0.12

Metabolite concentrations are reported in nmol/ng of tissue. GPCho: GlyceroPhosphoCholine, Pcho: PhosphoCholine.

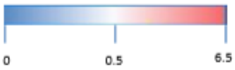

Figure S4. Global metabolic profiles of the BT69 tumors (diagnosis and relapse) and the effect of oxygen deprivation in the corresponding cell line.

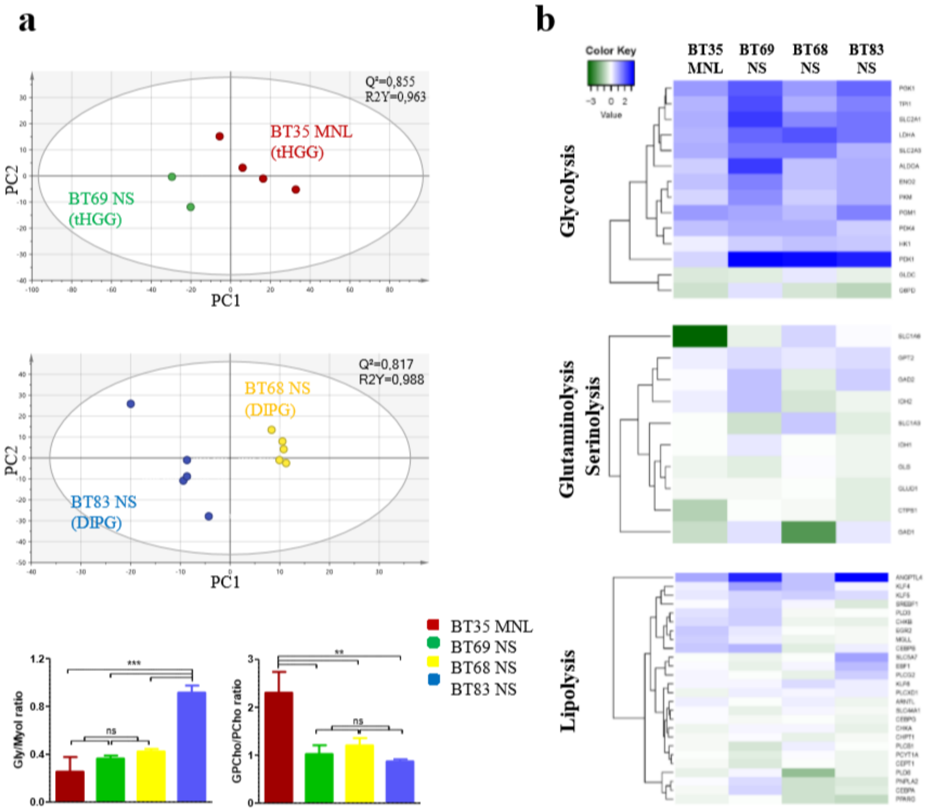

Figure S5. Metabolism assessment in cell lines established from *H3.3K28M*-mutated HGGs by 1H-HRMAS NMR spectroscopy and RNA sequencing.

a

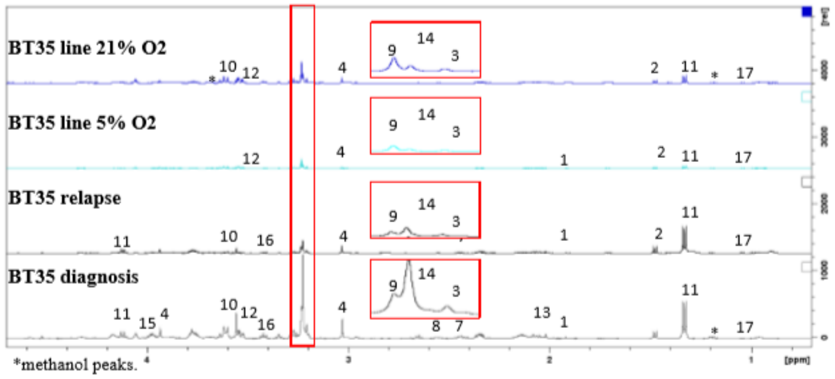

b

	Metabolites	Tumor BT35 diagnosis	Tumor BT35 relapse	PDCL BT35 5% O2	PDCL BT35 21% O2
1	Acetate	0,213	0,086	0,067	0,121
2	Alanine	1,469	0,999	0,301	0,449
3	Choline	0,814	0,149	0,078	0,182
4	Creatine	1,579	0,534	0,148	0,344
5	GABA	1,95	0,573	0,363	0,773
6	β-Glucose	0,237	0,2	0,176	0,497
7	Glutamate	4,418	1,657	0,504	1,188
8	Glutamine	2,298	0,711	0,037	0,123
9	GPCho	1,751	0,504	0,565	1,045
10	Glycine	1,274	0,19	0,116	0,317
11	Lactate	6,438	3,41	0,586	1,368
12	MyoI	5,961	0,9	0,895	2,44
13	NAA	0,505	0,233	0,055	0,12
14	PCho	3,409	0.694	0.271	0.491
15	Serine	6,472	1,22	0,454	0,681
16	Taurine	2,215	0,602	0,195	0,712
17	Valine	0,202	0,187	0,067	0,105
	GPCho/Pcho (9/14)	0.513	0.726	2.128	2.084
	Gly/MyoI (10/12)	0.213	0.211	0.129	0.129

Metabolite concentrations are reported in nmol/ng of tissue. GPCho: GlyceroPhosphoCholine, Pcho: PhosphoCholine.

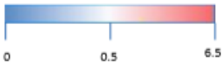

Figure S6. Global metabolic profiles of the BT35 tumors (diagnosis and relapse) and the effect of oxygen deprivation in the corresponding cell line.

a

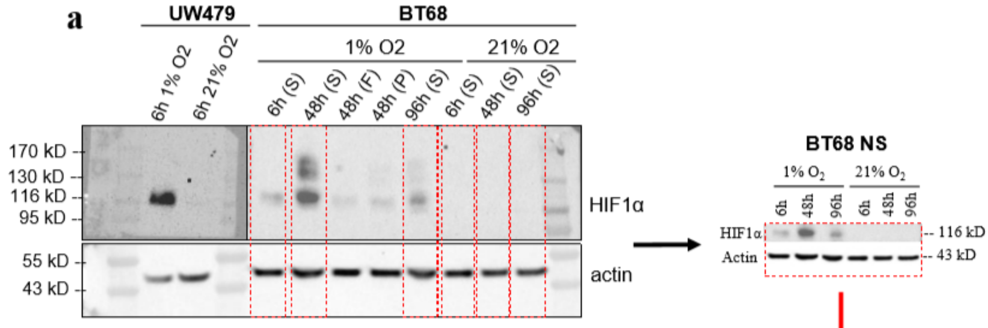

	Actin	HIF1α	HIF1α/actin
1%O2_6h (S)	6217140.00	461072.00	0.0742
1%O2_48h (S)	7419210.00	1590340.00	0.2144
1%O2_48h (F)	7127790.00	236180.00	0.0331
1%O2_48h (P)	6879800.00	255420.00	0.0371
1%O2_96h (S)	6471450.00	536047.00	0.0828
21%O2_6h (S)	5849800.00	0	0
21%O2_48h (S)	4994570.00	0	0
21%O2_96h (S)	5059920.00	0	0

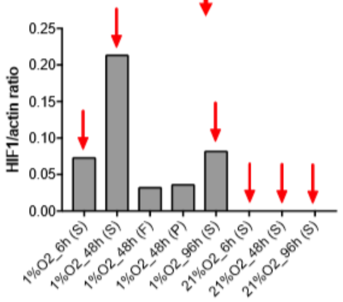

b

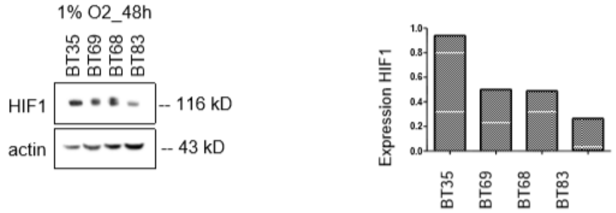

Figure S7. HIF-1α expression in normoxic and hypoxic cultures of patient-derived HGGs.

a b

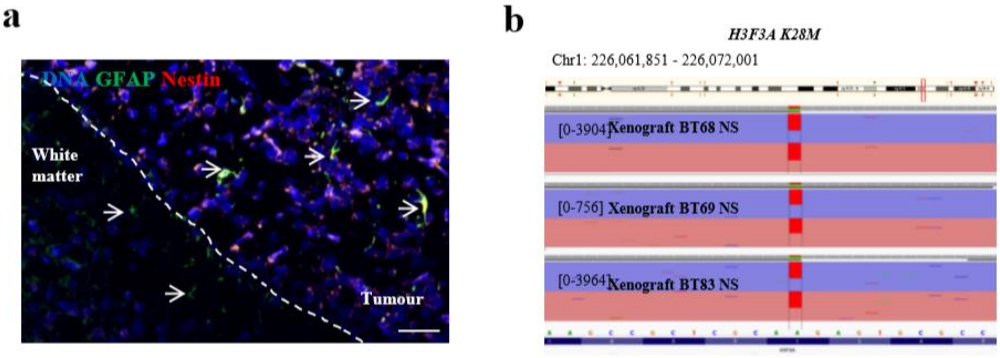

c

	Alu repeats (% human DNA)			
	< 0,1	0,1-10	10-50	> 50
Control	X			
BT35 MNL	X			
BT68 MNL	X			
BT83 MNL	X			
BT69 MNL	X			
BT68 NS				X
BT83 NS			X	
BT69 NS		X		

Figure S8. Tumor development following intracranial xenografts of neurosphere-patient derived cell lines.

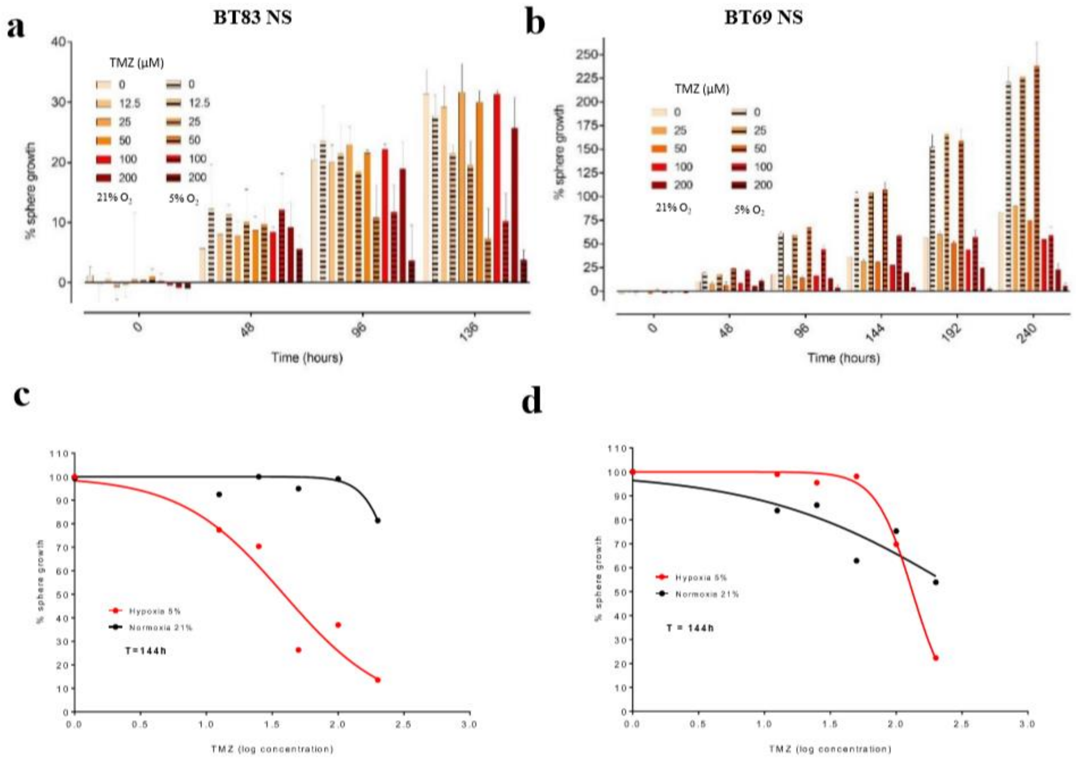

Figure S9. Oxygen-mediated response of PDCLs to Temozolomide.
